# Aids to Diagnosis in Nasal Disease

**Published:** 1881-12

**Authors:** Harrison Allen


### AIDS TO DIAGNOSIS IN NASAL DISEASE.

In making a diagnosis of nasal disease considerable difficulty is acknowledged. The nasal chambers are intricate in their outlines, the apertures permitting inspection are small, and the various structures seen therethrough are foreshortened to the eye.

### TO EXAMINE THE NASAL CHAMBERS.

The following rules have been framed with the object in view of simplifying the examination and to properly interpret what is seen.

1st. Bring the shoulders forward and throw the head far back. Insert the speculum. The under surface of the middle turbinated bone will be seen at its anterior part, provided no undue narrowing of the chamber exists. In the same position, inspect the chink between the anterior end of the middle turbinated bone and the outer wall of the vestibule.* Make such use of the probe as may be indicated to ascertain the condition of the mucous membrane, and the nature of the points of contact (if such should exist) between the median and lateral walls.

2d. The shoulders to be in the same position as in the preceding. The speculum being in place, bring the head slightly forward. The middle turbinated bone now passes from the field, and the ethmo-vomerine sutural projection, if such exists, becomes visible. When it is present, the posterior portion of the middle turbinated bone,

* By vestibule is meant the interior of the external nose.

the ethmo-vomerine projection, and the superior curved portion of the inferior turbinated bone are occasionally seen to be in contact with one another. No obstruction to breathing need follow upon this arrangement, provided the inferior portion of the chamber remains open. In other examples, the parts just mentioned are separated from one another, when a black curvilinear chink exists between the inferior turbinated bone and the septum. If this chink does not exist, by reason of the pressure of the sides against one another, distress is very apt to be acknowledged. During treatment the chink can be defined at the time the patient reports improvement. In yet another group of nasal chambers the parts in question are remote from one another, and the lower portion of the wall of the sphenoid sinus is seen, together with the upper portion of the choana.

3d. The shoulders still being forward, the head is brought into a horizontal position. The space between the sides of the inferior turbinated bone and the septum is now seen through the speculum. If the chamber is capacious, and the anterior osseous aperture large, the plane of the opening of the inferior meatus is visible and situated about half way up the outer side of the field. If, however, the parts are everywhere contracted, the plane of the inferior meatus orifice is not seen, or, if it is seen, its upper portion is only discernible.

Such inspection as is practicable, of the middle meatus, can be made either in the second or third positions.

4th. Preserving the forward position of the shoulders, as before, bring the head well down on the chest. Insert a small-calibred speculum and push it as far inward to the lower portion of the anterior osseous aperture as is possible. The floor of the nasal chamber is now seen at its anterior part. The chink between the anterior end of the inferior turbinated bone and the floor, and the floor of the vestibule itself, are all visible. This field is frequently obstructed by an outgrowth from the septum, either maxillary or vomerine in nature. Should hypertrophy of the inferior turbinated bone exist, the chink between the bone and floor of the nose is obliterated.

### TO DETECT THE SIGNIFICANCE OF TRACTION BANDS.

The history of recurrent obstruction from angiose turgescence can be confirmed by the presence of cobweb like threads of mucus stretching across the chamber from the lateral to the median wall. The following explanation is ventured upon: The surfaces once in contact have separated, but give evidence of their former position by these traction bands.

TO DETECT OBSTRUCTION.

Close the mouth and the nose of the opposite side ; then bring the shoulders forward and throw the head back. Request the patient to breathe. If the sound is sniffling, especially if the act be associated with adduction of the lateral wall of the external nose above the wing, obstruction exists. Then insert Zaufal's speculum in the premaxillary portion of the nose. Repeat the breathing-test. If the sniffling is now relieved, obstruction of the vestibule or the premaxillary region may be diagnosed. Should the sniffling persist, push the speculum into the maxillary portion, and in succession to the palatal, renewing the test of breathing in each instance ; or, the small rubber speculum being in position, raise suspicious folds of mucous membrane with a probe and request the patient to breathe. Such improvement of breathing after thus pressing aside the turgescent folds furnishes a clue to the nature and location of the obstruction. The presence of mucus in the nasal chamber can be detected by its motion forward and backward during respiration, when conducted in the manner above described.

TO PREPARE A NASAL CHAMBER FOR EXAMINATION WHERE GREAT NARROWING EXISTS FROM CONGESTION OF THE MEMBRANES.

Apply a primary current of electricity, from two to six cells. The cathode should be placed over the cheek below the infra-orbital foramen, and the anode on the nape of the neck, or at the mastoid fossa. After the current has passed for five minutes the patient will announce the fact that the obstruction is relieved. Inspection can now be made of the deeper parts with ease. Should the obstruction not yield to this test, a diagnosis of infiltration of the tissues may be made. An excessively angiose condition of the membranes accompanied with hyperæsthesia is rarely seen, which will not yield to the current. These applications are not only aids to diagnosis, but are often in themselves curative. Obstruction not due to turgescence or infiltration may be proved to be osseous by the probe.

TO DETERMINE THE CONDITION OF THE MEMBRANES BY THE REFLECTION OF LIGHT THEREFROM.

A moist surface yields a broad, brilliant reflection. A dry one yields a diffused, dull reflection, which at the same time that it is diffused is broken up into minute points of light.

A mammillated moist surface will throw off multiple reflections, but a uniformly convex surface will throw a single large pencil of light.

TO EXAMINE THE CELLS OF THE NASAL MUCOUS MEMBRANE.

A double angulated probe passed into the normally constituted space between the lower turbinated bone and the septum, and drawn forward at the same time that it is pressed firmly against one of the sides, can be withdrawn, bringing with it a drop of the mucus of the region through which it has passed. This drop, when examined with the microscope, will show characteristic epithelial cells with active cilia. The cells are deformed in outline, without cilia, and otherwise changed, in mild forms of atrophic degeneration of the turbinated bones; deformed, without cilia, and excessively granulated in the infiltration of syphilis; or, absent, in angiose turgescence and in advanced forms of atrophic degeneration. Angiose turgescence exists in hay fever and some forms of catarrh, associated with hyperæsthesia simulating hay fever. In such diseases the cells are not easily detached, and cannot be found in the mucus.

For clinical purposes the nasal region may be divided into the premaxillary, the maxillary, and the palatal portion. If sections be made by sawing the skull in frontal (transverse vertical) planes at the lines of sutural union on the hard palate, viz: between the premaxillæ and the maxillæ, and between the maxillæ and the palatal bones, subdivisions of the nasal chamber are secured which embrace more or less natural regions. The premaxillary portion includes the vestibule and the nasal chambers proper, so far as to embrace the anterior ends of the turbinated bones. The upward extension of the section would answer to the anterior border of the anterior of the cerebral fossa. The maxillary portion includes the turbinated bones within the point last mentioned and the hinder ends. Palatal portion includes the hinder ends of the turbinated bones and the region extending thence to the posterior nares.—*Dr. Harrison Allen in Am. Specialist.*

